# Supplementary material for: Neutralization capacity of antibodies elicited through homologous or heterologous infection or vaccination against SARS-CoV-2 VOCs
Source: Nat Commun. 2022 Jul 4;13:3840. doi: 10.1038/s41467-022-31556-1 (PMC9253337; doi:10.1038/s41467-022-31556-1)
Supplement: Supplementary file 1 — Supplementary Information [file 41467_2022_31556_MOESM1_ESM.pdf]

	B.1	Alpha	Beta	Gamma	Delta	Zeta	Omicron
**Name**	hCoV-19/Switzerland/GE-SNRCI-29943121/2020	hCoV-19/Switzerland/20 12212272/2020	hCoV-19/Switzerland/GE-33128281/2021	hCoV-19/Switzerland/GE-33115015/2021	hCoV-19/Switzerland/GE-33896105/2021	hCoV-19/Switzerland/GE-32966260/2021	hCoV-19/Switzerland/VD-HUG-36221084/2021
**Gisaid accession ID**	EPI_ISL_414019	EPI_ISL_2131446	EPI_ISL_981782	EPI_ISL_981707	EPI_ISL_1811202	EPI_ISL_897700	EPI_ISL_7605546
**Clade**	G	GRY	GH	GR	GK	G	GRA
**Pango lineage**	B.1	B.1.1.7	B.1.351	P.1	AY.122	P.2	BA.1

Table S1. Patient sample information from which virus isolates were obtained.

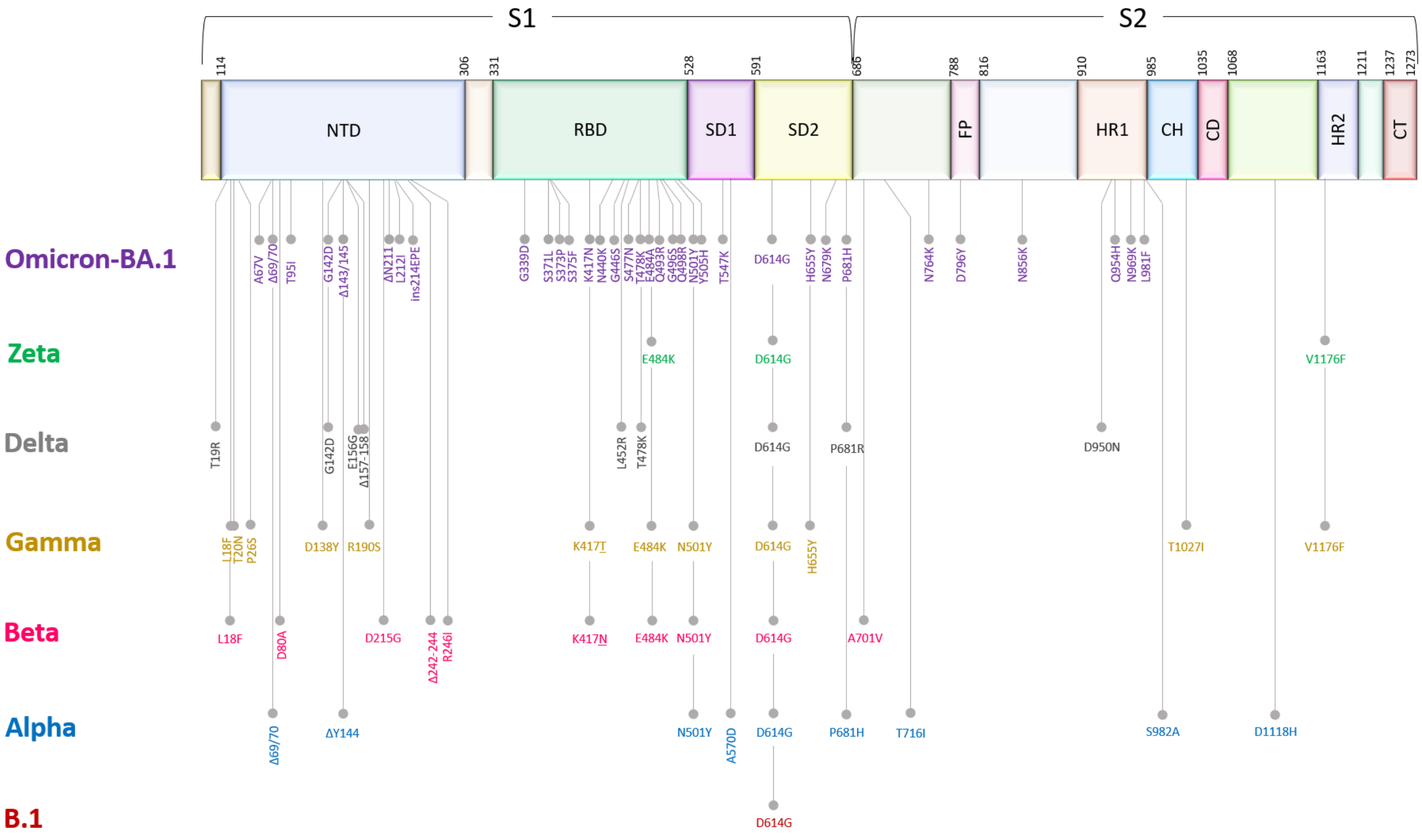

Fig S1. Domain organization and mutations of the SARS-CoV-2 Spike protein of different variants used in this study.

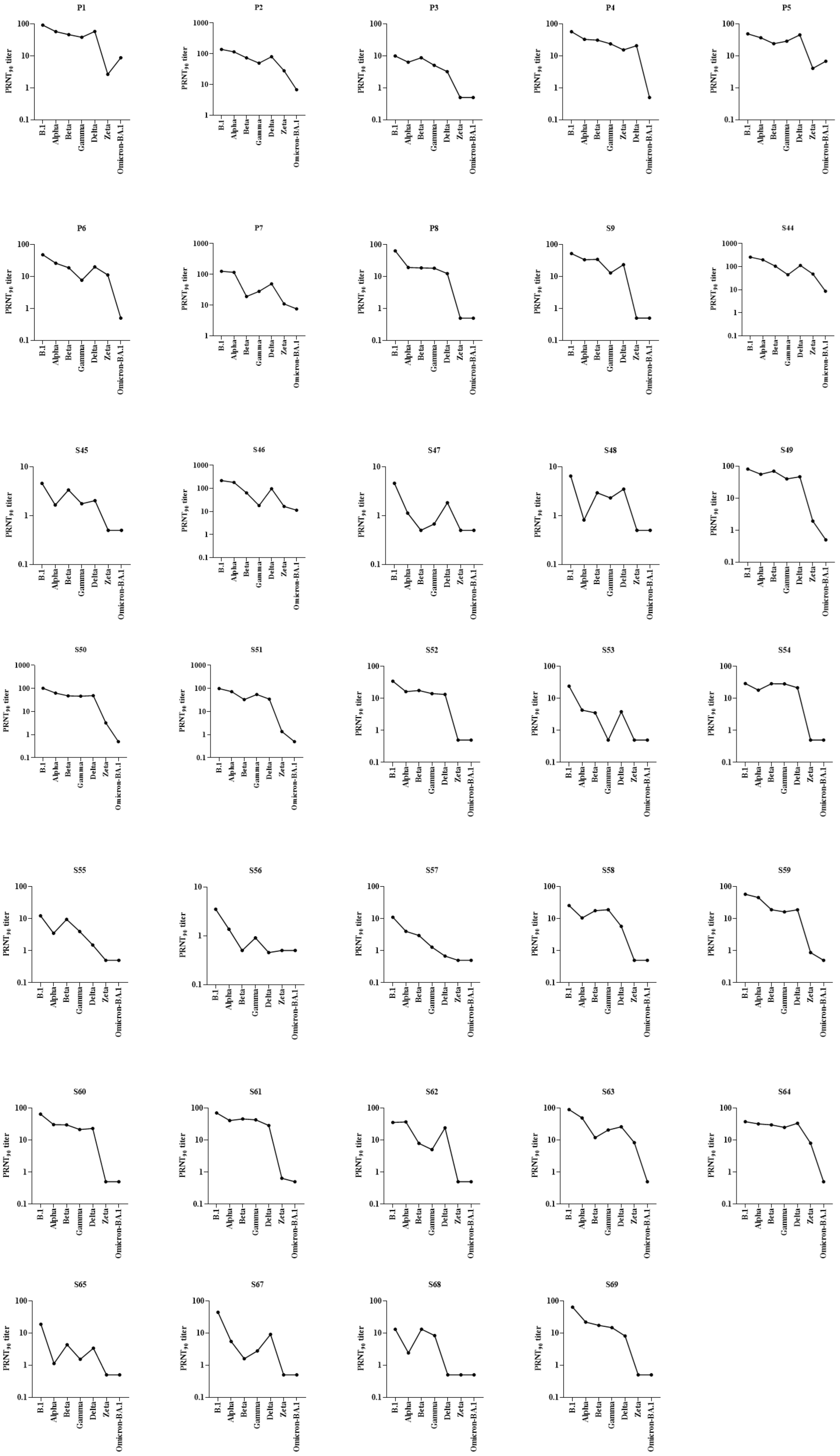

Fig S2. Escape of SARS-CoV-2 variants from preVOC elicited immunity. PRNT90 titers against SARS-CoV-2 variants (B.1, Alpha, Beta, Gamma, Delta, Zeta and Omicron-BA.1) determined using convalescent pre-VOC plasma/sera. Source data are provided as a Source Data file.

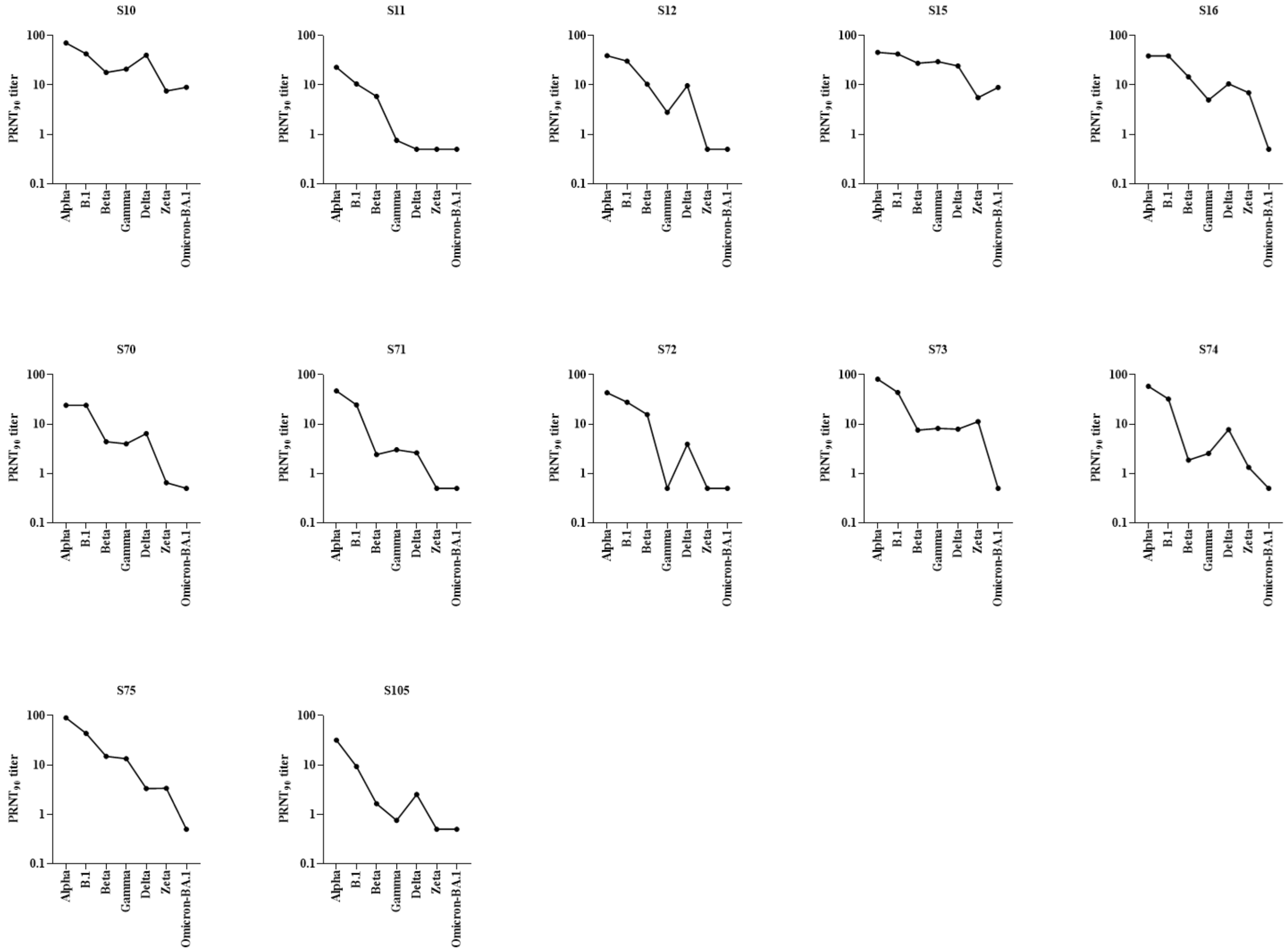

Fig S3. Escape of SARS-CoV-2 variants from Alpha elicited immunity. PRNT90 titers against SARS-CoV-2 variants (Alpha, B.1, Beta, Gamma, Delta, Zeta and Omicron-BA.1) determined using convalescent Alpha sera. Source data are provided as a Source Data file.

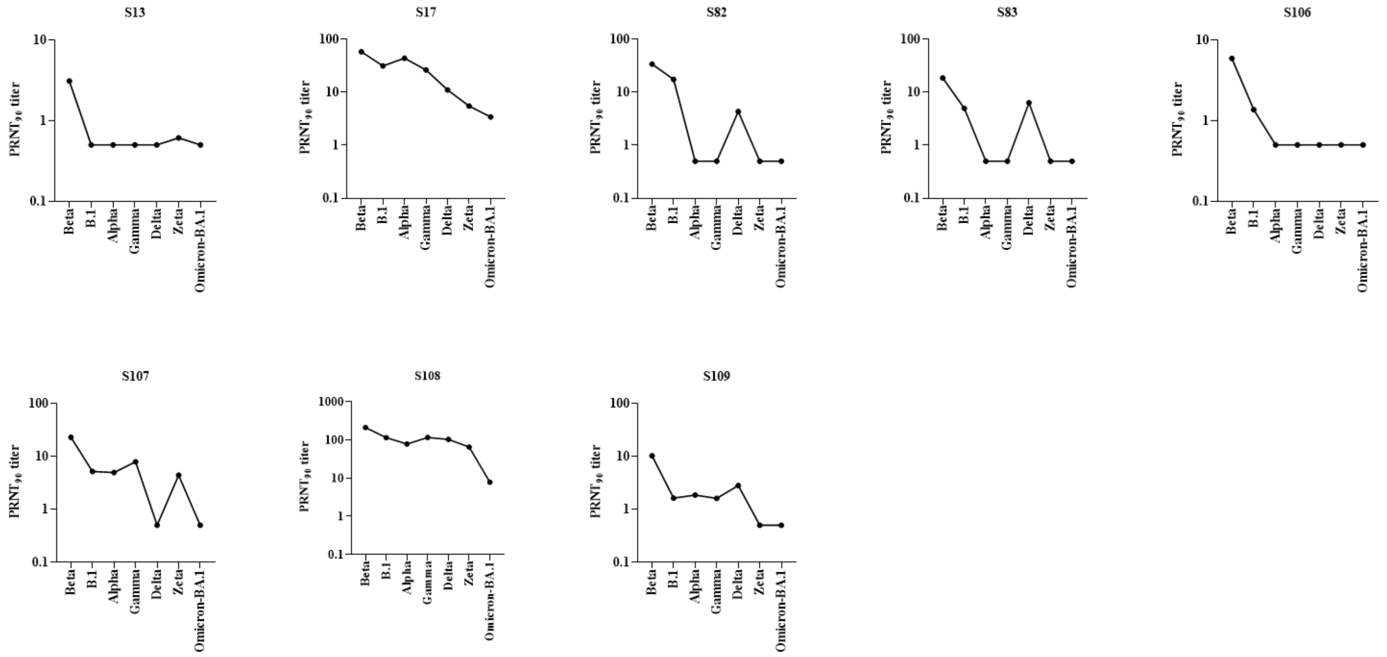

Fig S4. Escape of SARS-CoV-2 variants from Beta elicited immunity. PRNT90 titers against SARS-CoV-2 variants (Beta, B.1, Alpha, Gamma, Delta, Zeta and Omicron-BA.1) determined using convalescent Beta sera. Source data are provided as a Source Data file.

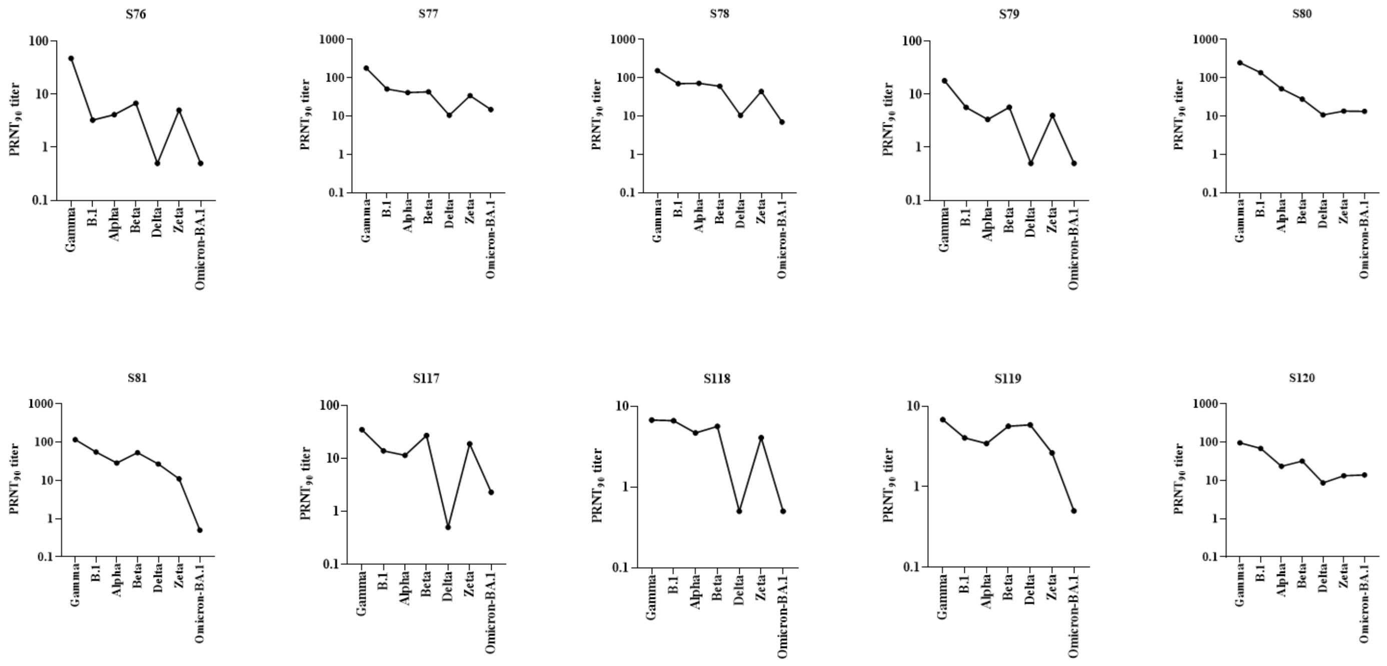

Fig S5. Escape of SARS-CoV-2 variants from Gamma elicited immunity. PRNT90 titers against SARS-CoV-2 variants (Gamma, B.1, Alpha, Beta, Delta, Zeta and Omicron-BA.1) determined using convalescent Gamma sera. Source data are provided as a Source Data file.

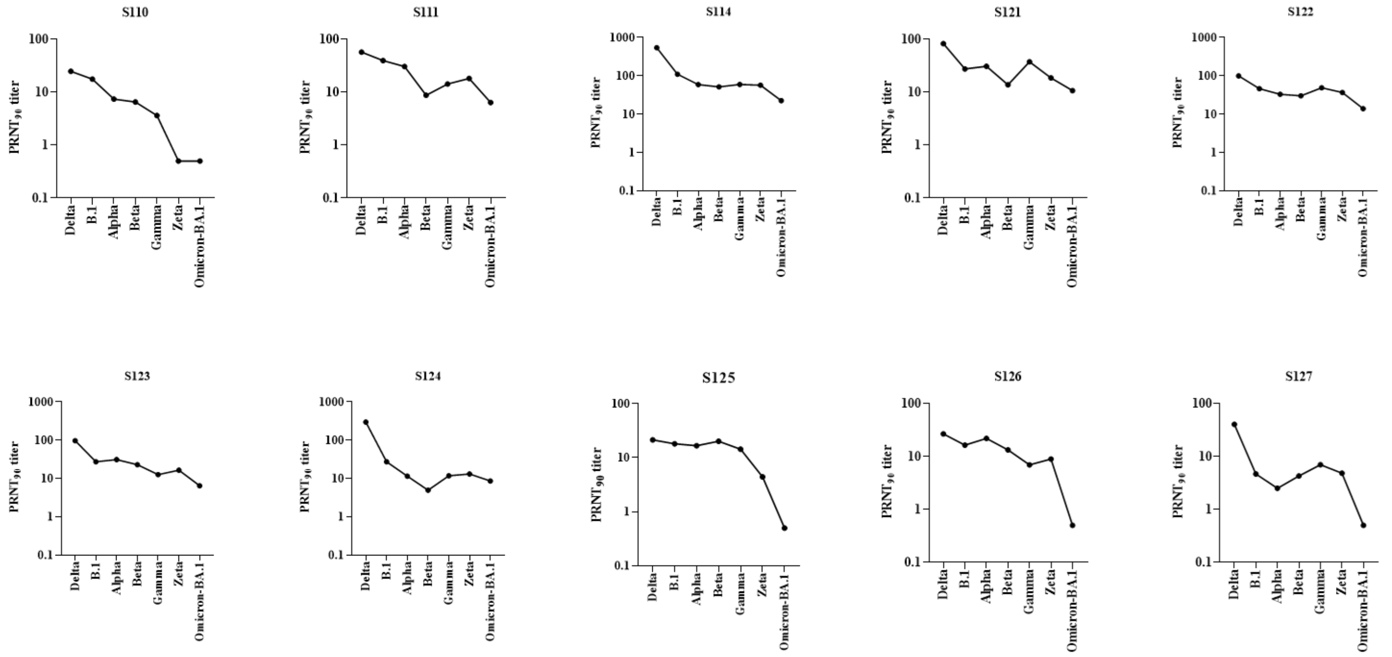

Fig S6. Escape of SARS-CoV-2 variants from Delta elicited immunity. PRNT90 titers against SARS-CoV-2 variants (Delta, B.1, Alpha, Beta, Gamma, Zeta and Omicron-BA.1) determined using convalescent Delta sera. Source data are provided as a Source Data file.

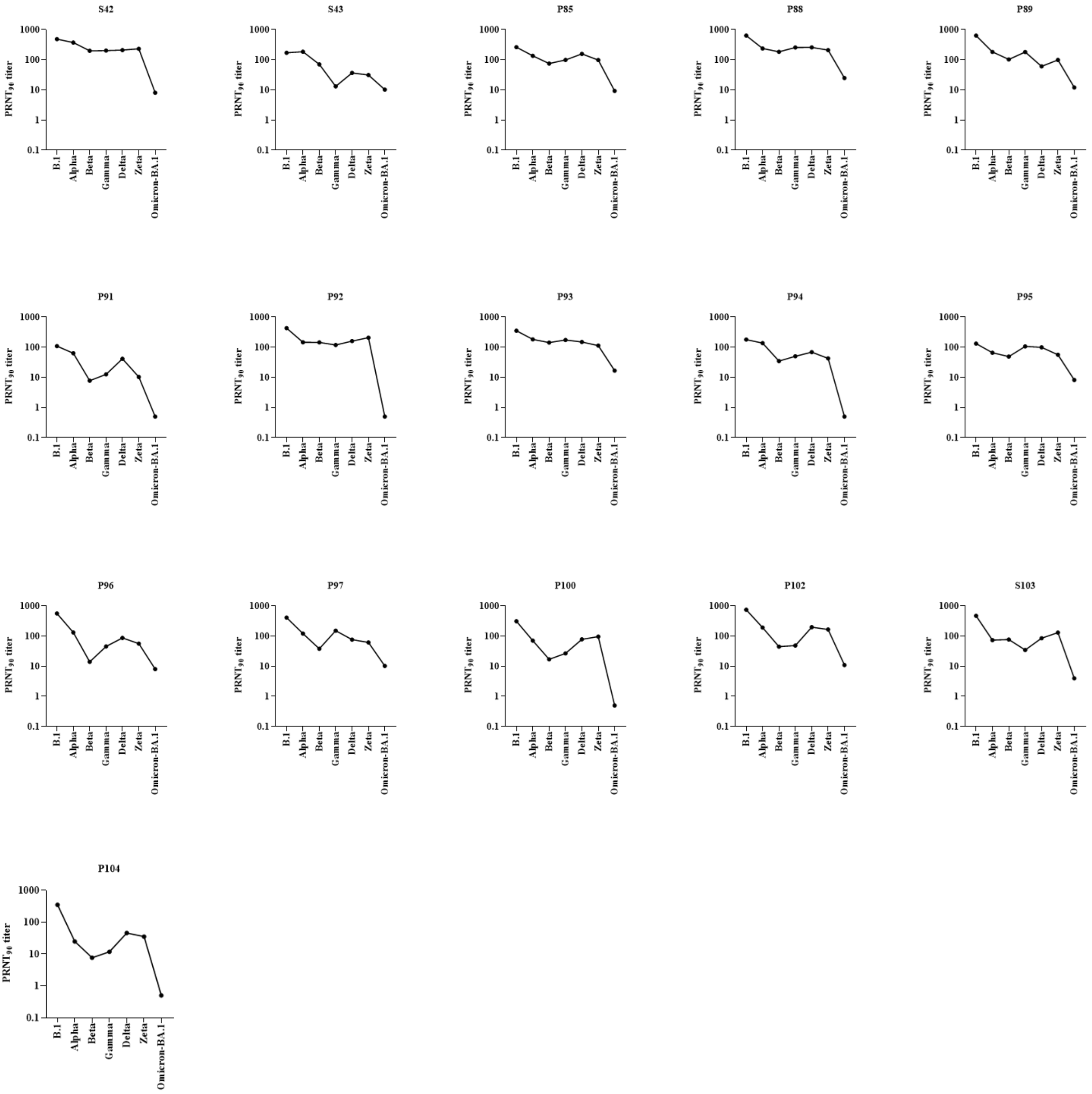

Fig S7. Escape of SARS-CoV-2 variants from double-dose mRNA vaccination elicited immunity. PRNT90 titers against SARS-CoV-2 variants (B.1, Alpha, Beta, Gamma, Delta, Zeta and Omicron-BA.1) determined using sera/plasma from individuals with double-dose mRNA vaccination. Source data are provided as a Source Data file.

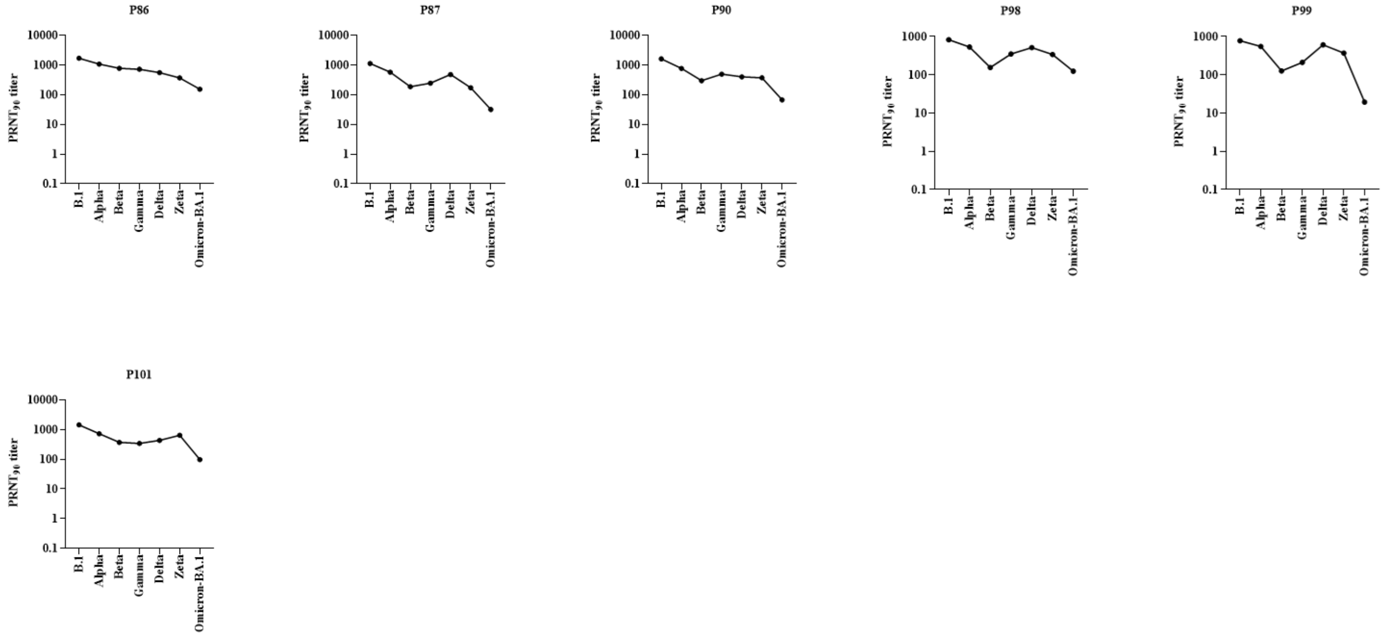

Fig S8. Escape of SARS-CoV-2 variants from prior SARS-CoV-2 infection followed by double-dose mRNA vaccination elicited immunity. PRNT90 titers against SARS-CoV-2 variants (B.1, Alpha, Beta, Gamma, Delta, Zeta and Omicron-BA.1) determined using plasma from individuals with prior SARS-CoV-2 infection followed by double-dose mRNA vaccination. Source data are provided as a Source Data file.

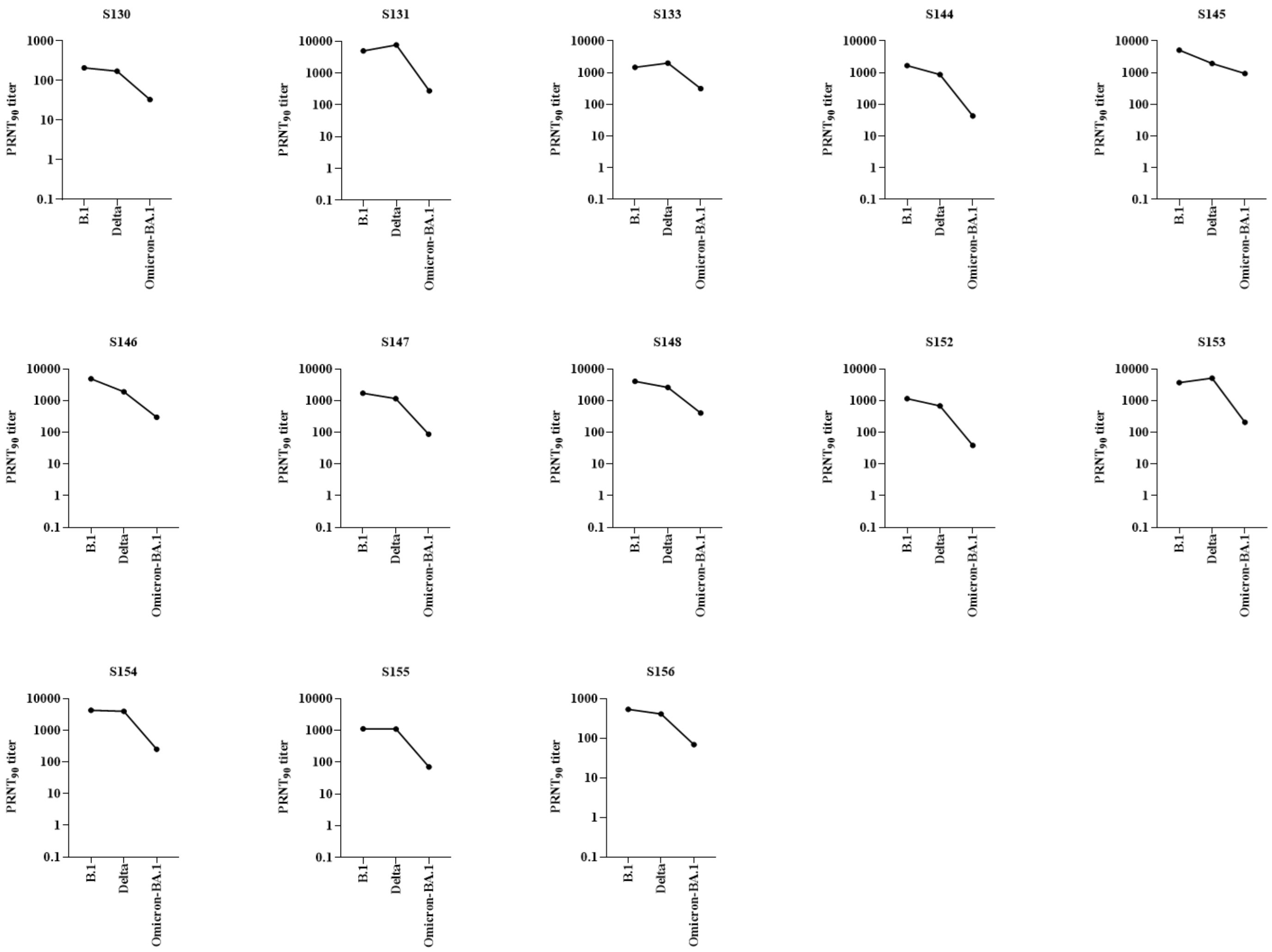

Fig S9. Escape of SARS-CoV-2 variants from Delta breakthrough infection elicited immunity. PRNT90 titers against SARS-CoV-2 variants (B.1, Delta and Omicron-BA.1) determined using sera from Delta breakthrough infection of double-vaccinated individuals. Source data are provided as a Source Data file.

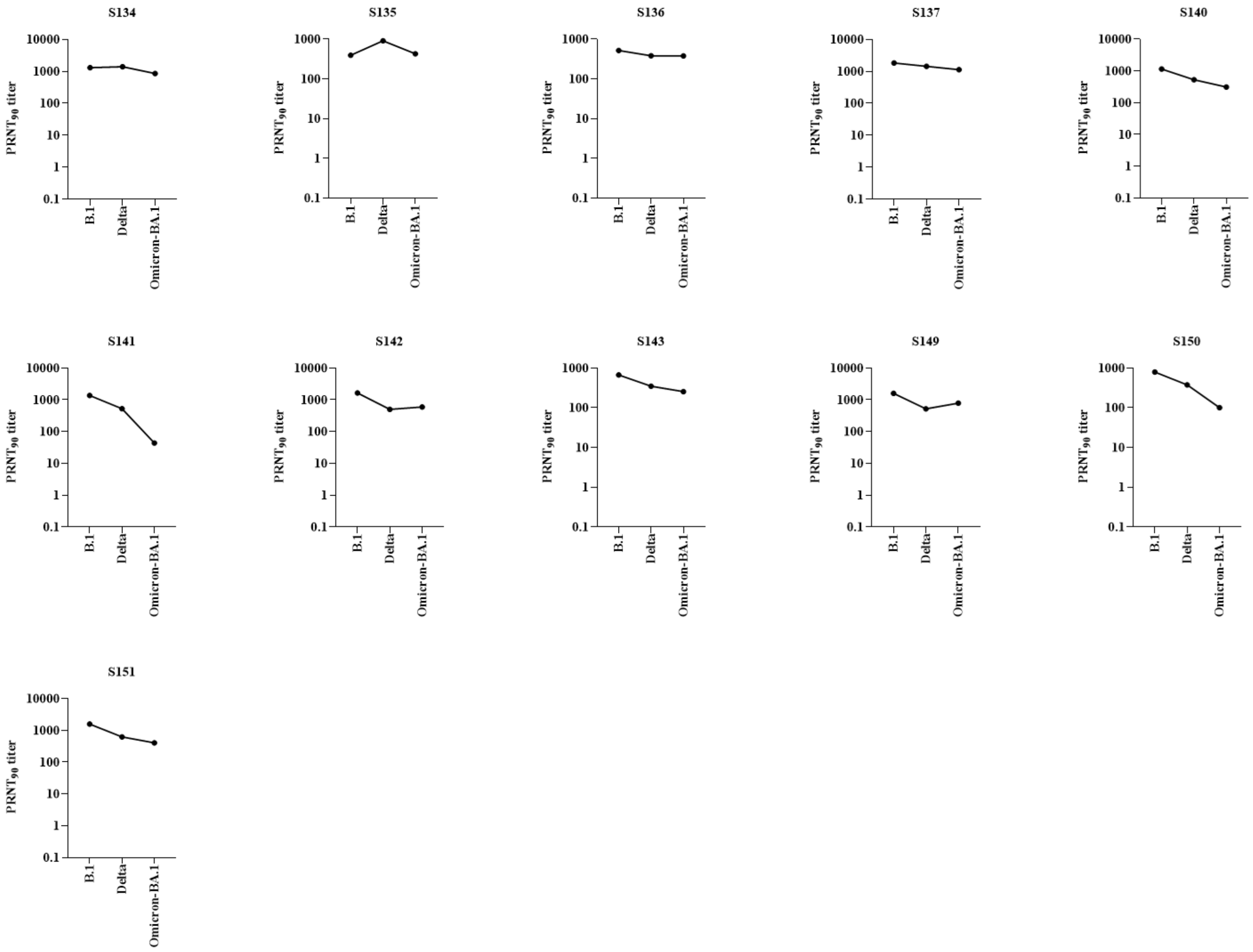

Fig S10. Escape of SARS-CoV-2 variants from Omicron-BA.1 breakthrough infection elicited immunity. PRNT90 titers against SARS-CoV-2 variants (B.1, Delta and Omicron-BA.1) determined using sera from Omicron-BA.1 breakthrough infection of double and single vaccinated individuals. Source data are provided as a Source Data file.

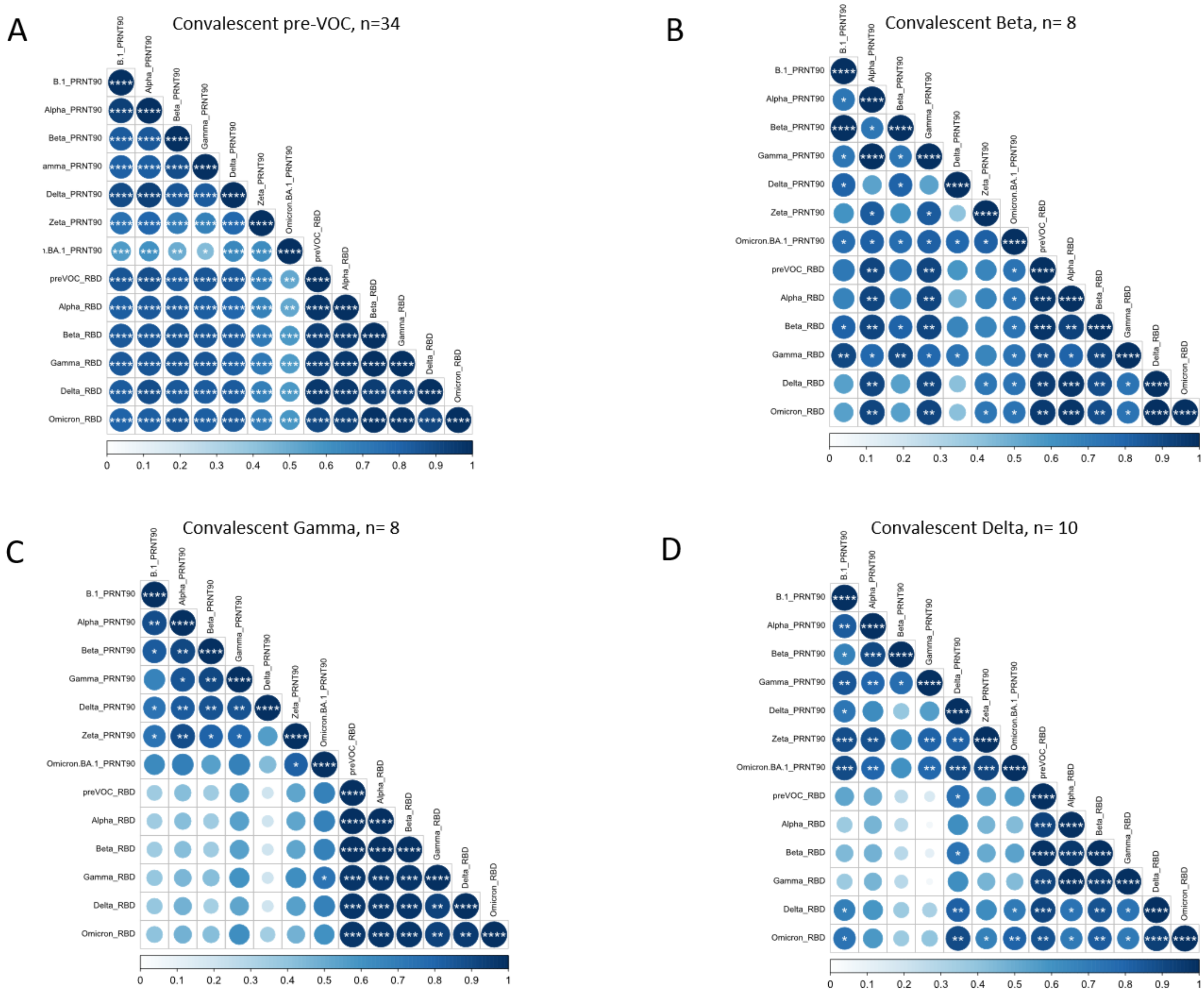

Fig S11. Spearman correlation analysis between RBD-binding IgG titers and neutralizing titer ($PRNT_{90}$) against 6 SARS-CoV-2 strains (B.1, Alpha, Beta, Gamma, Delta and Omicron-BA.1) from infection-derived convalescent samples. (A-D) Cohorts of convalescent specimens that are derived from individuals infected with (A) early-pandemic SARS-CoV-2 (pre-VOC), (B) Beta (C) Gamma (D) Delta. Asterisks indicate level of significance (two-sided), if no asterisks are shown, correlation is not significant, * *p* value < 0.05, ** *p* value < 0.01, *** *p* value < 0.001, **** *p* value < 0.0001. Colors indicate Spearmans rho correlation coefficient. Source data are provided as a Source Data file.

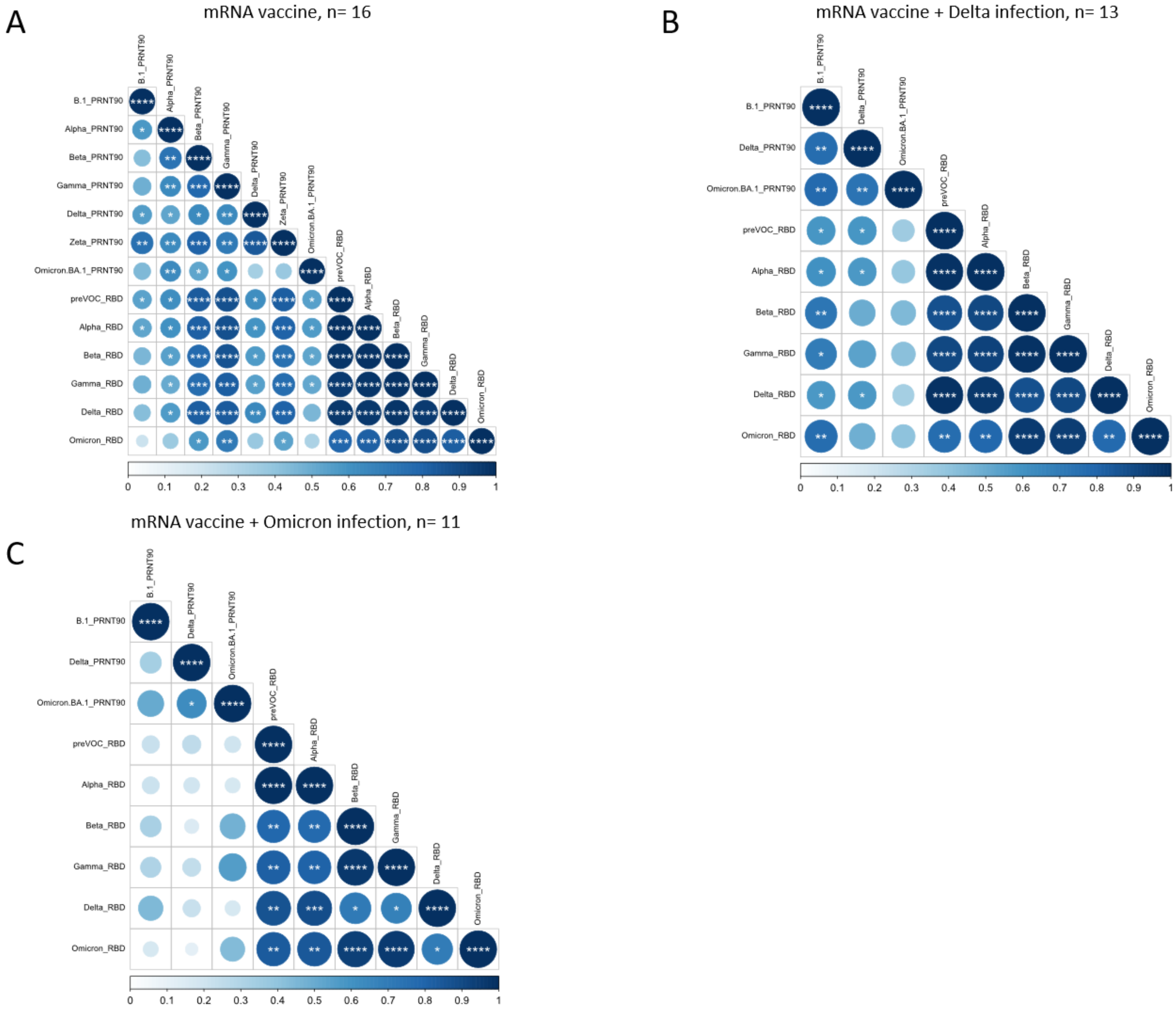

Fig S12. Spearman correlation analysis between RBD-binding IgG titer and neutralizing titer ($PRNT_{90}$) against 6 SARS-CoV-2 strains (B.1, Alpha, Beta, Gamma, Delta and Omicron-BA.1) from post-vaccine and combined post-vaccine/infection-derived samples. (A-C) Cohorts consist of individuals with (A) double-dose mRNA vaccination, (B) Delta breakthrough infection of double-vaccinated individuals and (C) Omicron-BA.1 breakthrough infection following double (n=8) and single (n=3) mRNA vaccination. Asterisks indicate level of significance (two-sided), if no asterisks are shown, correlation is not significant, * *p* value < 0.05, ** *p* value < 0.01, *** *p* value < 0.001, **** *p* value < 0.0001. Colors indicate Spearmans rho correlation coefficient. Source data are provided as a Source Data file.
