# Supplementary material for: Neutralization capacity of antibodies elicited through homologous or heterologous infection or vaccination against SARS-CoV-2 VOCs
Source: Nat Commun. 2022 Jul 4;13:3840. doi: 10.1038/s41467-022-31556-1 (PMC9253337; doi:10.1038/s41467-022-31556-1)
Supplement: Supplementary file 3 — Reporting Summary [file 41467_2022_31556_MOESM3_ESM.pdf]

nature portfolio

Corresponding author(s): Benjamin Meyer, Isabella Eckerle

Last updated by author(s): May 24, 2022

Reporting Summary

Nature Portfolio wishes to improve the reproducibility of the work that we publish. This form provides structure for consistency and transparency in reporting. For further information on Nature Portfolio policies, see our Editorial Policies and the Editorial Policy Checklist.

Statistics

For all statistical analyses, confirm that the following items are present in the figure legend, table legend, main text, or Methods section.

n/a	Confirmed	
☐	☒	The exact sample size (*n*) for each experimental group/condition, given as a discrete number and unit of measurement
☐	☒	A statement on whether measurements were taken from distinct samples or whether the same sample was measured repeatedly
☐	☒	The statistical test(s) used AND whether they are one- or two-sided<br>*Only common tests should be described solely by name; describe more complex techniques in the Methods section.*
☐	☒	A description of all covariates tested
☒	☐	A description of any assumptions or corrections, such as tests of normality and adjustment for multiple comparisons
☐	☒	A full description of the statistical parameters including central tendency (e.g. means) or other basic estimates (e.g. regression coefficient) AND variation (e.g. standard deviation) or associated estimates of uncertainty (e.g. confidence intervals)
☐	☒	For null hypothesis testing, the test statistic (e.g. *F*, *t*, *r*) with confidence intervals, effect sizes, degrees of freedom and *P* value noted<br>*Give P values as exact values whenever suitable.*
☒	☐	For Bayesian analysis, information on the choice of priors and Markov chain Monte Carlo settings
☒	☐	For hierarchical and complex designs, identification of the appropriate level for tests and full reporting of outcomes
☐	☒	Estimates of effect sizes (e.g. Cohen's *d*, Pearson's *r*), indicating how they were calculated

Our web collection on statistics for biologists contains articles on many of the points above.

Software and code

Policy information about availability of computer code

Data collection	Data collection was done using Excel 2019
Data analysis	Data analysis was done using GraphPad Prism software version V 9.1.0, R Statistical Software version 4.1.1 and online tool: https://acmacs-web.antigenic-cartography.org/

For manuscripts utilizing custom algorithms or software that are central to the research but not yet described in published literature, software must be made available to editors and reviewers. We strongly encourage code deposition in a community repository (e.g. GitHub). See the Nature Portfolio guidelines for submitting code & software for further information.

Data

Policy information about availability of data

All manuscripts must include a data availability statement. This statement should provide the following information, where applicable:

- Accession codes, unique identifiers, or web links for publicly available datasets
- A description of any restrictions on data availability
- For clinical datasets or third party data, please ensure that the statement adheres to our policy

The minimum datasets presented in this article are included as a Source Data file in this manuscript. The personal information related to the participants presented in this article are available under restricted access because no consent has been obtained from participants for public sharing of anonymized datasets. Requests to access the personal information of participants should be directed to corresponding authors.

March 2021

Human research participants

Policy information about studies involving human research participants and Sex and Gender in Research.

Reporting on sex and gender	For the study design, sex was based on self-reporting.<br>Median Male/Female (M/F) ratio for convalescent individuals infected with ancestral SARS-CoV-2 was: M/F = 20/14, for Alpha it was: M/F = 8/4, for Beta it was: M/F = 2/6, for Gamma it was:M/F = 4/6 or for Delta it was: M/F = 4/6, for double vaccinated individuals it was: M/F = 4/12, for double vaccinated individuals with prior infection it was: M/F = 3/3 and fo individuals after breakthrough infections with Delta it was: M/F = 5/8 or for Omicron-BA.1 it was: M/F =7/4.<br>The information related to personal data of participants can not be published because no consent has been obtained for sharing individual level data.
Population characteristics	We included a total of 120 individuals previously infected with SARS-CoV-2, a SARS-CoV-2 variant of concern or who were vaccinated or vaccinated and infected later on with Delta or Omicron-BA.1. This included 34 individuals infected with SARS-CoV-2 in 2020 (age mean: 31), 12 individuals infected with the Alpha variant (age mean: 51), 8 individuals infected with the Beta variant (age mean: 42), 10 individuals infected with the Gamma variant (age mean: 44) and 10 individuals infected with the Delta variant (age mean: 42). Of the vaccinated cohort, this included 16 individuals vaccinated twice (age mean: 52) and 6 participants that were found to be infected by the presence of anti-nucleocapsid antibodies in their post-vaccine specimen (age mean: 46). In addition, we have included 13 2x-vaccinated individuals with a Delta breakthrough infection (age mean: 43) and 8 2x-vaccinated individuals with an Omicron-BA.1 breakthrough (age mean: 38). 3 individuals were vaccinated only once and had an Omicron-BA.1 infection (age mean: 28).
Recruitment	Selection of sera/plasma was usually from the first identified cases in our routine diagnostic without specific selection processes. All participants had mild Covid-19. Indeed, we only chose outpatient and not hospitalized to avoid any bias. Specimens from infected individuals: Specimens from patients infected in 2020 ongoing prospective observational study at the Geneva University Hospital (HUG) (Ethics approval number: CCER 2020-00516). In addition, anonymized left-over plasma from apheresis collection of plasma (all collected in 2020) were available under the general informed consent of the University Hospitals of Geneva. Patients infected with a variant of concern were first tested for SARS-CoV-2 as part of routine diagnostic testing of the hospital. We contacted them, randomly, in the re-convalescent period to ask for their willingness of participating in the study and collected blood from them, same for breakthrough infections (Ethics approval number: CCER 2020-02323). Specimens from vaccinated individuals were collected as part of a prospective observational study (Ethics approval number: CCER ICOVax 2021-00430.
Ethics oversight	Cantonal Ethics Committee (CCER) at the University Hospitals of Geneva approved the protocol and the research plan for this study

Note that full information on the approval of the study protocol must also be provided in the manuscript.

Field-specific reporting

Please select the one below that is the best fit for your research. If you are not sure, read the appropriate sections before making your selection.

- [x] Life sciences
- [ ] Behavioural & social sciences
- [ ] Ecological, evolutionary & environmental sciences

For a reference copy of the document with all sections, see nature.com/documents/nr-reporting-summary-flat.pdf

Life sciences study design

All studies must disclose on these points even when the disclosure is negative.

Sample size	120 serum/plasma samples. Sample size was restricted by availability of post-infection and/or characterized post-vaccination specimens
Data exclusions	No data was excluded
Replication	Observational studies, no randomization. PRNTS were determined in duplicates and all attempts at replication were successful. RBD-binding IgG antibody titers were determined only in one replicate due to the low volume of sera.
Randomization	Observational study, no randomization was done
Blinding	Observational study, no blinding was done.

Reporting for specific materials, systems and methods

We require information from authors about some types of materials, experimental systems and methods used in many studies. Here, indicate whether each material, system or method listed is relevant to your study. If you are not sure if a list item applies to your research, read the appropriate section before selecting a response.

Materials & experimental systems

n/a	Involved in the study	
x		Antibodies
	x	Eukaryotic cell lines
x		Palaeontology and archaeology
x		Animals and other organisms
x		Clinical data
x		Dual use research of concern

Methods

n/a	Involved in the study	
x		ChIP-seq
x		Flow cytometry
x		MRI-based neuroimaging

Eukaryotic cell lines

Policy information about cell lines and Sex and Gender in Research

Cell line source(s)	Vero-E6 cells (ATCC CRL-1586) received from Volker Thiel lab, Vero E6-TMPRSS provided by National Institute for Biological Standards and Controls and A549 overexpressing human ACE-2 were received from the laboratory of Mirco Schmolke. No commercial sources.
Authentication	Cell lines were not authenticated recently.
Mycoplasma contamination	Cells were not tested for mycoplasma contamination.
Commonly misidentified lines (See ICLAC register)	Commonly misidentified lines have not been used in this study.
