# Supplementary material for: Neutralization capacity of antibodies elicited through homologous or heterologous infection or vaccination against SARS-CoV-2 VOCs
Source: Nat Commun. 2022 Jul 4;13:3840. doi: 10.1038/s41467-022-31556-1 (PMC9253337; doi:10.1038/s41467-022-31556-1)
Supplement: Supplementary file 4 — Source Data [file 41467_2022_31556_MOESM4_ESM.pdf]

Figure 1 (A-I)
Figure 2
Figure 3
Figures S2-S12

		PRNT90						
**Infecting virus/vaccine**	**Sera/Plasma**	**B.1**	**Alpha**	**Beta**	**Gamma**	**Delta**	**Zeta**	**Omicron BA.1**
pre-VOC	P1	92.23	57.73	46.4	38.44	58.4	2.648	8.788
pre-VOC	P2	138.3	115.3	73.67	49.37	80.05	27.93	6.768
pre-VOC	P3	9.984	6.378	8.8	5.097	3.224	0.5	0.5
pre-VOC	P4	57.28	33.02	31.15	23.94	20.81	15.43	0.5
pre-VOC	P5	49.13	37.12	24.22	28.97	45.65	4.082	6.809
pre-VOC	P6	47.88	26.03	18.79	7.67	20.03	11.16	0.5
pre-VOC	P7	125.6	115.6	19.04	28	49.04	10.92	7.5
pre-VOC	P8	63.7	19.25	18.64	18.29	12.42	0.5	0.5
pre-VOC	S9	52.35	33.64	34.43	12.89	23.47	0.5	0.5
pre-VOC	S44	257.6	196.6	104.2	44.34	112.8	47.73	8.587
pre-VOC	S45	4.577	1.647	3.359	1.755	2.028	0.5	0.5
pre-VOC	S46	217.9	181.7	63.76	17.98	96.41	16.56	11.38
pre-VOC	S47	4.605	1.126	0.5	0.6744	1.837	0.5	0.5
pre-VOC	S48	6.46	0.8145	2.932	2.304	3.481	0.5	0.5
pre-VOC	S49	81.97	56.38	70.46	40.5	47.09	1.928	0.5
pre-VOC	S50	102.3	62.82	47.59	46.09	48.86	3.228	0.5
pre-VOC	S51	99.1	72.67	33.07	54.67	34.43	1.363	0.5
pre-VOC	S52	34.31	16.31	17.68	14.11	13.33	0.5	0.5
pre-VOC	S53	24.11	4.301	3.548	0.5	3.833	0.5	0.5
pre-VOC	S54	29.15	18.06	28.73	28.4	21.47	0.5	0.5
pre-VOC	S55	12.24	3.505	9.483	3.963	1.5	0.5	0.5
pre-VOC	S56	3.501	1.36	0.5	0.9084	0.4528	0.5	0.5
pre-VOC	S57	11.06	4.02	2.978	1.287	0.6775	0.5	0.5
pre-VOC	S58	25.68	10.52	17.72	18.95	5.743	0.5	0.5
pre-VOC	S59	57.65	45.63	19.04	16.24	18.94	0.8722	0.5
pre-VOC	S60	64.24	30.29	29.85	21.29	23.04	0.5	0.5
pre-VOC	S61	70.03	40.58	45.46	42.88	28.37	0.6398	0.5
pre-VOC	S62	35.45	36.92	7.867	5.024	24.11	0.5	0.5
pre-VOC	S63	89.62	48.82	11.95	20.62	25.89	8.308	0.5
pre-VOC	S64	37.39	31.97	29.71	24.75	33.47	7.952	0.5
pre-VOC	S65	18.75	1.127	4.336	1.525	3.373	0.5	0.5
pre-VOC	S67	44.36	5.475	1.588	2.776	9.108	0.5	0.5
pre-VOC	S68	13.04	2.404	13.16	8.364	0.5	0.5	0.5
pre-VOC	S69	64.09	21.99	17.34	14.75	8.124	0.5	0.5
Alpha	S10	42.9	71.06	17.95	20.92	40.2	7.548	8.983
Alpha	S11	10.5	22.79	5.873	0.7548	0.5	0.5	0.5
Alpha	S12	30.46	39.28	10.41	2.788	9.669	0.5	0.5
Alpha	S15	42.59	45.81	27.65	29.69	24.34	5.539	8.919
Alpha	S16	38.71	38.69	14.6	4.96	10.57	6.979	0.5
Alpha	S70	24.04	24.01	4.394	3.999	6.428	0.6529	0.5
Alpha	S71	24.43	47.2	2.424	3.033	2.624	0.5	0.5
Alpha	S72	27.71	43.17	15.6	0.5	3.913	0.5	0.5
Alpha	S73	43.78	81.59	7.579	8.211	7.907	11.26	0.5
Alpha	S74	32.3	58.46	1.872	2.554	7.719	1.333	0.5
Alpha	S75	43.77	90.57	15.03	13.43	3.335	3.386	0.5
Alpha	S105	9.294	31.82	1.641	0.7554	2.534	0.5	0.5
Beta	S13	0.5	0.5	3.1	0.5	0.5	0.6101	0.5
Beta	S17	31.32	43.69	57.89	26.45	11.05	5.472	3.426
Beta	S82	17.38	0.5	33.9	0.5	4.325	0.5	0.5
Beta	S83	4.96	0.5	18.56	0.5	6.324	0.5	0.5
Beta	S106	1.245	0.5	5.907	0.5	0.5	0.5	0.5
Beta	S107	5.167	4.932	22.88	7.853	0.5	4.397	0.5
Beta	S108	114.6	77.82	209.7	115	103.1	64.66	7.777
Beta	S109	1.628	1.856	10.19	1.604	2.805	0.5	0.5
Gamma	S76	3.27	4.122	6.782	47.79	0.5	5.023	0.5
Gamma	S77	51.26	41.23	43.18	180.2	10.55	34.36	14.86
Gamma	S78	70.67	72.44	60.33	154	10.4	44.22	6.997
Gamma	S79	5.686	3.368	5.703	18.06	0.5	3.999	0.5
Gamma	S80	136.2	52.02	27.95	248	10.76	13.59	13.36
Gamma	S81	55.18	28.59	53.01	116.2	26.71	10.97	0.5
Gamma	S117	13.91	11.45	27.17	34.96	0.5	18.93	2.304
Gamma	S118	6.62	4.669	5.625	10.52	0.5	4.086	0.5
Gamma	S119	4.04	3.429	5.625	11.49	5.844	2.63	0.5
Gamma	S120	68.52	23.41	31.95	95.95	8.597	13.25	13.88
Delta	S110	17.68	7.392	6.518	3.618	24.72	0.5	0.5
Delta	S111	39.4	30.51	8.75	14.22	57.08	18.12	6.348
Delta	S114	109.8	59.1	51.33	59.47	541.2	56.78	22.46
Delta	S121	27.43	30.75	13.79	37.53	82.6	18.57	10.71
Delta	S122	46.19	32.95	29.99	48.96	99.75	36.84	13.85
Delta	S123	27.32	31.09	22.78	12.53	96.72	16.42	6.41
Delta	S124	27.32	11.45	4.9	11.7	295.8	13	8.522
Delta	S125	18.15	16.72	20.25	14.44	21.47	4.429	0.5
Delta	S126	16.4	21.9	13.33	6.94	26.71	8.958	0.5
Delta	S127	4.665	2.499	4.289	6.967	40.39	4.816	0.5
2x mRNA vaccine	S42	485.1	371.8	199.3	202	209	232.6	8.073
2x mRNA vaccine	S43	171	185.8	70.45	13.12	36.68	31.37	10.27
2x mRNA vaccine	P85	262.8	135.6	75.14	98.86	156.7	97.76	9.268
2x mRNA vaccine	P88	635.3	237.2	184.1	255.3	257.4	210	24.74

2x mRNA vaccine	P89	633.4	182.5	102.6	181.2	60.28	98.69	12.04
2x mRNA vaccine	P91	108.2	62.96	7.739	12.5	41.16	10.3	0.5
2x mRNA vaccine	P92	427.5	145.3	143.6	118.3	158.8	206.1	0.5
2x mRNA vaccine	P93	351.9	181.6	141.4	173.6	148.9	112.7	16.61
2x mRNA vaccine	P94	178.4	136.8	34.67	49.74	68.18	42.58	0.5
2x mRNA vaccine	P95	131.7	65.5	48.75	105.8	99.05	55.91	8.166
2x mRNA vaccine	P96	561.4	131	14.01	45.07	87.03	55.91	8.014
2x mRNA vaccine	P97	407.8	121	37.92	151	76.18	61.44	10.22
2x mRNA vaccine	P100	310	70.64	16.86	26.55	78.2	96.02	0.5
2x mRNA vaccine	P102	743	190.8	44.49	48.49	197.7	165.3	10.93
2x mRNA vaccine	P103	471.3	73.13	76.76	34.65	84.64	130.6	3.958
2x mRNA vaccine	P104	347.4	24.61	7.605	11.7	45.67	35.02	0.5
Prior infection + 2x mRNA vaccine	P86	1693	1075	778.1	717.2	556.2	367.6	153.8
Prior infection + 2x mRNA vaccine	P87	1128	576.9	185.6	244.4	479.3	172.7	31.87
Prior infection + 2x mRNA vaccine	P90	1605	764.6	296.8	495.4	400.9	372.2	67.1
Prior infection + 2x mRNA vaccine	P98	831	533.6	155.4	351.8	516.1	340.6	124.1
Prior infection + 2x mRNA vaccine	P99	778.9	553.2	126	210.8	609.4	368.7	19.37
Prior infection + 2x mRNA vaccine	P101	1434	726.7	371.6	340.1	432.9	639.2	97.42
2x vaccination + Delta breakthrough	S130	210.1	/	/	/	173.6	/	33.27
2x vaccination + Delta breakthrough	S131	4931	/	/	/	7590	/	274.7
2x vaccination + Delta breakthrough	S133	1468	/	/	/	2004	/	316.1
2x vaccination + Delta breakthrough	S144	1667	/	/	/	870.9	/	43.28
2x vaccination + Delta breakthrough	S145	5152	/	/	/	1953	/	942
2x vaccination + Delta breakthrough	S146	4831	/	/	/	1899	/	297.7
2x vaccination + Delta breakthrough	S147	1710	/	/	/	1148	/	86.87
2x vaccination + Delta breakthrough	S148	4057	/	/	/	2599	/	404.6
2x vaccination + Delta breakthrough	S152	1153	/	/	/	681	/	38.58
2x vaccination + Delta breakthrough	S153	3672	/	/	/	5068	/	205.6
2x vaccination + Delta breakthrough	S154	4242	/	/	/	3913	/	249
2x vaccination + Delta breakthrough	S155	1116	/	/	/	1092	/	70.65
2x vaccination + Delta breakthrough	S156	531.8	/	/	/	406	/	68.25
2x vaccination + Omicron breakthrough	S134	1301	/	/	/	1396	/	852.9
1x vaccination + Omicron breakthrough	S135	163.9	/	/	/	911.6	/	426.7
1x vaccination + Omicron breakthrough	S136	520.6	/	/	/	381.6	/	377.7
2x vaccination + Omicron breakthrough	S137	1821	/	/	/	1434	/	1131
2x vaccination + Omicron breakthrough	S140	1145	/	/	/	529.8	/	311.7
1x vaccination + Omicron breakthrough	S141	1368	/	/	/	521	/	43.5
2x vaccination + Omicron breakthrough	S142	1646	/	/	/	497.2	/	593.7
2x vaccination + Omicron breakthrough	S143	668.6	/	/	/	347.8	/	254.8
2x vaccination + Omicron breakthrough	S149	1591	/	/	/	520.3	/	783.2
2x vaccination + Omicron breakthrough	S150	789.7	/	/	/	375.6	/	100.2
2x vaccination + Omicron breakthrough	S151	1577	/	/	/	623.7	/	402.3

		**MSD-panel 22**					
**Infecting virus/vaccine**	**Sera/Plasma**	**S1 RBD**	**RBD Alpha**	**RBD Bêta**	**RBD Gamma**	**RBD Delta**	**RBD Omicron**
pre-VOC	P1	5918.097721	5941.066889	3134.942752	4418.843045	5391.569441	1050.159494
pre-VOC	P2	89859.64246	68998.23306	12807.35882	20813.13533	34972.39372	4759.372891
pre-VOC	P3	3390.80098	3467.557298	885.0332811	1397.45404	2559.37528	328.1539607
pre-VOC	P4	5804.953946	5312.28826	1459.023501	2462.831276	4817.774186	620.6871829
pre-VOC	P5	9984.877373	7488.66247	4401.616344	6459.517677	29344.90767	1589.500917
pre-VOC	P6	2250.699001	2032.724785	1127.877863	1727.461473	2094.245609	460.5245045
pre-VOC	P7	7543.807986	6877.249489	3506.811504	5133.929222	6864.136526	1223.173078
pre-VOC	P8	5345.962862	4129.88088	1022.000844	1700.561161	2753.809681	249.7239983
pre-VOC	S9	2562.511231	2452.862653	1120.718321	1616.384917	2585.123623	552.6123685
pre-VOC	S44	57276.60076	58314.99257	31106.17648	61226.26728	68885.29323	26208.90841
pre-VOC	S45	1085.453734	1003.896416	319.3113104	445.0864659	830.4936576	132.4398482
pre-VOC	S46	34141.93711	29987.71854	11622.39842	19002.51446	24733.59222	3422.711323
pre-VOC	S47	1082.092979	852.3056213	194.7262554	262.0717509	797.6261363	50.95031339
pre-VOC	S48	2109.500186	1998.025047	1055.294015	1425.417078	804.0980632	507.0921309
pre-VOC	S49	17757.82735	14158.53293	5923.552381	9206.054647	14746.07189	1852.550822
pre-VOC	S50	17635.18355	15241.39237	6175.289123	9840.003151	15959.62501	1876.233141
pre-VOC	S51	15746.58869	13660.33415	5197.799526	8000.485571	12601.15285	2078.475269
pre-VOC	S52	5525.445031	4882.990329	1741.785198	3566.33845	4594.814368	704.2900451
pre-VOC	S53	3256.943887	2541.214877	564.7562943	915.4126991	2431.143215	188.9436087
pre-VOC	S54	9489.067518	7064.462066	2182.105987	3422.159154	5872.203712	680.5414095
pre-VOC	S55	2233.766719	2297.682293	937.9502539	1495.231867	1466.770382	364.2237478
pre-VOC	S56	741.3110838	555.3890471	133.3060828	192.9471441	452.1808627	48.20220082
pre-VOC	S57	1033.019271	949.4253285	131.9961042	235.8508506	807.5585924	34.28649502
pre-VOC	S58	2246.319104	1955.803509	750.3446958	1254.155932	1750.168267	323.1222439
pre-VOC	S59	10096.62039	8750.114428	3456.377039	5224.446208	9360.309417	905.6748548
pre-VOC	S60	4763.064188	4222.248461	1787.387079	2872.749231	3769.027018	645.3938668
pre-VOC	S61	8535.296716	8021.15641	2320.929289	4025.538453	6596.423453	884.1905336
pre-VOC	S62	2967.909845	1140.692459	341.992168	558.3057418	2873.211452	109.2923141
pre-VOC	S63	6529.965542	5460.174754	1361.671319	2155.359254	4128.089772	416.2205544
pre-VOC	S64	4865.96611	2012.453983	614.9717334	929.1655717	1403.734773	238.3937525
pre-VOC	S65	876.6007202	979.9323866	146.2046839	244.0117741	812.6052384	70.17897278
pre-VOC	S67	1384.684549	490.1479084	154.8999832	250.5819478	333.3746379	54.98169637
pre-VOC	S68	1101.752583	1125.34626	556.3926297	922.3559178	1125.954095	49.11818617
pre-VOC	S69	6765.053082	6515.442134	1347.220433	1964.2856	4800.92373	371.413462
Alpha	S10	/	/	/	/	/	/
Alpha	S11	/	/	/	/	/	/
Alpha	S12	2988.900781	5372.583014	1120.718321	1907.729239	2469.514337	445.3135502
Alpha	S15	90694.67372	151645.036	26551.49984	45397.18718	48783.18309	11946.55083
Alpha	S16						
Alpha	S70	2230.251981	3307.139042	809.7988941	1271.852261	1607.654839	255.1486581
Alpha	S71	3300.353251	3960.534137	1361.458819	2223.650133	2725.077824	565.4965731

Alpha	S72	/	/	/	/	/	/
Alpha	S73	/	/	/	/	/	/
Alpha	S74	10772.5176	14835.15596	3581.952913	5846.588993	8037.032548	1034.825728
Alpha	S75	4646.949022	6745.103658	1795.36172	2627.067266	3161.374992	479.3637863
Alpha	S105	4737.82655	9854.448986	721.1209798	1195.797532	1940.083031	207.807483
Beta	S13	43.34414099	69.47722942	75.49603438	118.2995862	37.86999532	24.59214804
Beta	S17	6976.145138	10223.90834	12364.67666	16524.39473	5078.629795	923.2022239
Beta	S82	/	/	/	/	/	/
Beta	S83	/	/	/	/	/	/
Beta	S106	77.83891129	115.42674	162.0898493	211.6471744	76.30951706	36.4827098
Beta	S107	900.8575258	1479.116759	1504.601372	2080.994429	565.3974227	263.9878155
Beta	S108	5773.973236	5407.885686	4043.02482	5619.386689	4886.385144	566.0503905
Beta	S109	501.6838234	906.5367488	591.0825037	898.6793496	307.4697941	69.82321516
Gamma	S76	2112.574043	2339.26617	2192.040228	4367.95427	2589.230429	543.3453325
Gamma	S77	17800.01556	25647.07466	21709.25584	43469.35243	23575.28555	5776.953657
Gamma	S78	23396.8889	30558.95081	28376.51591	48124.79053	28935.93992	7228.985324
Gamma	S79	802.2554178	1055.629576	802.3165063	1291.320384	510.1425951	178.7708745
Gamma	S80	/	/	/	/	/	/
Gamma	S81	261.2538787	378.0555682	412.7347196	571.1204447	255.0188703	77.90292153
Gamma	S117	413.9847388	546.5013229	489.5716312	1283.735134	340.596624	69.64850676
Gamma	S118	629.2809543	803.3020465	790.7706674	1159.134529	803.9467865	152.1068577
Gamma	S119	/	/	/	/	/	/
Gamma	S120	3915.653295	5738.737963	5592.627533	8925.609644	3902.433631	1679.377699
Delta	S110	2870.32331	2650.400883	1259.048275	2074.740027	8371.158806	689.561386
Delta	S111	8988.406101	7226.687047	3396.400802	5391.98749	23548.02382	1255.537865
Delta	S114	60140.14836	55486.33367	22009.11171	38615.06009	260457.9126	12606.58468
Delta	S121	1883.289728	1973.835545	953.1918646	1525.753187	4226.590319	638.1262333
Delta	S122	5469.937061	5155.053427	2347.561606	3947.030933	10672.34045	1443.114974
Delta	S123	9032.192174	7880.024422	4435.293779	6884.532281	32729.9487	1413.268871
Delta	S124	10606.5091	8847.682856	4587.907774	7511.650658	30068.24843	1758.585606
Delta	S125	213.8493377	237.029842	96.70944984	157.0447722	753.3002497	65.55549765
Delta	S126	7661.555578	27182.26625	4090.085518	10990.17546	2384.296178	356.3269809
Delta	S127	519.4979899	546.3369089	283.5347705	470.7525887	1133.708648	91.55322681
2x mRNA vaccine	S42	111226.8531	100736.3447	52608.7186	86358.428	96828.41356	31725.4786
2x mRNA vaccine	S43	35897.57035	34580.61642	13971.40853	25847.05892	41235.74123	7984.237999
2x mRNA vaccine	P85	94853.86572	78566.30229	36566.65148	56333.71113	82821.97792	12535.31157
2x mRNA vaccine	P88	142531.0311	133785.8354	69452.01046	105053.2059	134574.9033	26812.02435
2x mRNA vaccine	P89	104741.1869	98727.83565	39352.09138	64575.31656	77549.10168	13874.36795
2x mRNA vaccine	P91	19510.13578	17854.98993	7404.50805	11240.28662	17576.29946	2506.391341
2x mRNA vaccine	P92	100094.5468	83434.1075	34156.90315	49908.70677	87020.76359	10656.33611
2x mRNA vaccine	P93	161841.5588	154489.5386	54681.94317	100877.9515	133181.5444	31605.87474
2x mRNA vaccine	P94	62903.79375	56296.49395	29777.48747	45246.74011	58377.78738	14672.87895
2x mRNA vaccine	P95	46751.4304	40349.0492	18866.81541	34854.49528	50343.20639	10972.11578
2x mRNA vaccine	P96	24906.32031	23962.91268	11529.09247	18847.23578	25229.14034	5358.204304
2x mRNA vaccine	P97	98094.27838	90359.71567	41579.59966	67637.61924	85179.05918	23583.44158
2x mRNA vaccine	P100	58215.17885	54230.8161	27877.22121	46271.50224	64535.21929	14884.98413
2x mRNA vaccine	P102	67800.77958	58650.08575	25393.93838	42703.71723	55174.05778	7141.502256
2x mRNA vaccine	P103	73922.08095	70036.83484	31759.11656	58467.16866	65050.09233	13063.74361
2x mRNA vaccine	P104	37402.8443	36843.59911	16255.2825	26907.59741	35275.17515	10275.06872
Prior infection + 2x mRNA vaccine	P86	122499.0856	118477.1315	54206.2386	72970.7131	105611.6325	23227.26142
Prior infection + 2x mRNA vaccine	P87	88127.30457	85336.6378	38752.68752	60982.47243	85663.2351	22635.83649
Prior infection + 2x mRNA vaccine	P90	107846.4851	100736.3447	48673.63227	70166.15053	91059.52184	17715.21849
Prior infection + 2x mRNA vaccine	P98	76740.77262	75076.63695	37779.72671	60886.51273	74123.90648	19442.37855
Prior infection + 2x mRNA vaccine	P99	93242.34595	86246.30152	40197.87211	69158.80297	87030.91859	19509.10406
Prior infection + 2x mRNA vaccine	P101	146966.7569	139491.3416	78189.35266	116742.5151	140805.6907	36390.5323
2x vaccination + Delta breakthrough	S130	38119.01979	35440.72868	18636.35453	28501.72266	37875.22083	8460.714157
2x vaccination + Delta breakthrough	S131	500864.0246	433343.3526	212645.8785	347212.914	437093.2805	128129.3656
2x vaccination + Delta breakthrough	S133	165554.0219	157855.9737	91677.14315	135291.6384	145543.6229	25477.55595
2x vaccination + Delta breakthrough	S144	299451.5361	253972.5349	107602.6217	180718.4676	253899.4714	40843.22053
2x vaccination + Delta breakthrough	S145	485419.0725	432328.3378	215283.8164	310532.0784	395759.0978	69657.52259
2x vaccination + Delta breakthrough	S146	390320.7019	343204.1242	139468.2867	226686.9946	341122.2895	57630.33831
2x vaccination + Delta breakthrough	S147	379870.6494	330402.861	104011.5307	167044.0462	341637.7341	39785.61579
2x vaccination + Delta breakthrough	S148	592727.098	544627.6745	202233.4964	310669.0247	540601.3426	55927.64434
2x vaccination + Delta breakthrough	S152	1402802.841	938931.4872	237579.5905	384420.5911	688193.5453	127022.3218
2x vaccination + Delta breakthrough	S153	516375.8062	450189.7699	112440.9305	181409.6618	437164.5649	33184.74965
2x vaccination + Delta breakthrough	S154	511080.4258	406775.4902	170273.2151	256365.8911	448051.249	61974.89356
2x vaccination + Delta breakthrough	S155	101087.0133	92639.63194	51094.24725	76433.46499	89224.0275	15813.78749
2x vaccination + Delta breakthrough	S156	56336.16595	53423.75292	28927.10084	46173.15895	50070.3831	13566.23065
2x vaccination + Omicron breakthrough	S134	153555.3305	150628.6539	87413.28441	135892.1657	121988.7871	30495.25036
1x vaccination + Omicron breakthrough	S135	177251.5716	160711.4509	80352.06428	141222.245	100994.9587	32682.70321
1x vaccination + Omicron breakthrough	S136	226323.1176	210642.086	93419.59187	146279.8309	113189.4102	44389.57287
2x vaccination + Omicron breakthrough	S137	244284.4602	238117.6519	136777.6281	220471.1717	138375.3858	50413.75469
2x vaccination + Omicron breakthrough	S140	263135.0817	268686.5522	147157.8205	217462.3157	152826.4814	62000.39217
1x vaccination + Omicron breakthrough	S141	208178.8019	186994.1314	40579.72615	64142.01459	143040.4096	10290.0877
2x vaccination + Omicron breakthrough	S142	239896.1388	238385.4531	133392.2789	201884.7335	152524.1015	58822.9758
2x vaccination + Omicron breakthrough	S143	97579.56182	91092.4875	61467.70079	92453.84472	76845.58018	21570.69258
2x vaccination + Omicron breakthrough	S149	172760.9769	160359.7843	96201.53962	149473.0108	128488.4654	41722.8087
2x vaccination + Omicron breakthrough	S150	39203.22337	36008.82529	19039.05754	29668.32569	36084.99707	8112.016967
2x vaccination + Omicron breakthrough	S151	140884.021	131868.543	82571.44412	128175.9561	94730.72182	21507.62647
